# Prevalence and factors associated with HIV infection among injection drug users at methadone clinics in Taipei, Taiwan

**DOI:** 10.1186/1471-2458-14-682

**Published:** 2014-07-04

**Authors:** Yung-Feng Yen, Muh-Yong Yen, Ting Lin, Lan-Huei Li, Xiao-Ru Jiang, Pesus Chou, Chung-Yeh Deng

**Affiliations:** 1 Section of Infectious Diseases, Taipei City Hospital, Taipei City Government, 145, Zhengzhou Rd., Datong Dist., Taipei 10341, Taiwan; 2 Community Medicine Research Center and Institute of Public Health, National Yang-Ming University, 155, Section 2, Ni-Long Street, Taipei 11221, Taiwan; 3 Department of Disease Control and Prevention, Taipei City Hospital, Taipei City Government, 145, Zhengzhou Rd., Datong Dist., Taipei 10341, Taiwan; 4 Institute of Hospital and Health Care Administration, National Yang-Ming University, 155, Section 2, Ni-Long Street, Taipei 11221, Taiwan

**Keywords:** HIV, Taiwan, Injection drug use, Methadone

## Abstract

**Background:**

Methadone treatment was introduced in Taiwan in 2006 as a harm-reduction program for injection drug users (IDUs), among whom HIV was endemic. We examined the association of HIV serostatus with demographic characteristics, substance use, and sexual behaviors among IDUs at methadone clinics in Taipei, Taiwan.

**Methods:**

During 2012–2013, IDUs at methadone clinics in Taipei were recruited to complete a risk assessment interview and undergo serologic testing for HIV infection. Correlates of HIV infection were identified by multivariate logistic regression.

**Results:**

Of the 827 eligible participants, 85.9% were male, median age was 45 years, and mean years of injecting was 18.0 (range 1–56). The prevalence of HIV infection was 17.7%. In multivariate analysis, HIV infection was significantly associated with age ≤45 years (adjusted odds ratio [AOR] = 1.62, 95% confidence interval [CI] 1.01–2.62), being divorced (AOR = 1.67, 95% CI 1.06–2.62), deriving the majority of income during the previous 6 months from temporary jobs or other noncriminal sources (AOR = 1.53, 95% CI 1.02–2.30), unstable housing during the previous 6 months (AOR = 1.47, 95% CI 1.003–2.15), higher number of incarcerations (AOR = 1.14, 95% CI 1.03–1.26), and a history of overdose (AOR = 1.51, 95% CI 1.01–2.28).

**Conclusions:**

Taiwanese IDUs at methadone clinics have a relatively high HIV prevalence, which was associated with younger age and history of overdose. It is imperative to educate IDUs’ about HIV transmission, particularly for the younger and overdosed IDUs.

## Background

Injection drug users (IDUs) are susceptible to blood-borne infectious diseases such as human immunodeficiency virus (HIV) infection. In 2008, there were an estimated 15.9 million IDUs globally, of whom 18.9% were infected with HIV [1]. In Asia in the same year, HIV prevalence among IDUs was approximately 16.0% overall (range 1.0% to 42.6%) [1]. IDUs have poor adherence to HIV screening and treatment [2], which complicates HIV control. It is therefore vital to investigate factors associated with HIV infection among IDUs in order to help design measures that are effective in curtailing HIV spread among this vulnerable population.

In Taiwan, HIV infection was first identified in a male foreigner in 1984 and later among homosexuals, heterosexuals, IDUs, pregnant women, those receiving blood transfusions, and hemophilia patients requiring infusion of clotting factor [3]. The Taiwan Department of Health has provided HIV-infected persons with free antiretroviral therapy since 1997 [4]. The number of newly reported HIV/AIDS cases continued to increase rapidly every year: from 860 cases in 2003 to 1520 cases in 2004 to 3380 cases in 2005. The percentage of these cases attributed to IDUs also increased markedly, from 9.3% in 2003 to 40.9% in 2004 to 71.6% in 2005 [3]. To curb this surge in HIV among IDUs, the Taiwan Centers for Disease Control (CDC) began a harm reduction program in 2006, including syringe exchange services for IDUs and methadone therapy for heroin addicts. At the end of 2012, the number and percentage of newly reported HIV cases attributable to IDUs had decreased significantly, to 81 cases and 3.6%, respectively. Presently in Taiwan, IDUs are the second most frequent source of HIV infection, accounting for 27.6% of all reported HIV cases [3].

IDUs are socially marginalized people and are hard to reach in the community. Studies that attempted to recruit IDUs from one or more treatment or correctional centers were able to enroll only a limited number of research subjects or HIV-positive cases [5-8]. Recently, two studies that analyzed HIV surveillance data might have underestimated HIV prevalence among IDUs, because information on HIV status was collected many months or years before the subjects entered into correctional institutions or methadone programs [9,10]. Another study of IDUs, which did not use Western blot to confirm HIV status, might have overdiagnosed the condition [11]. Moreover, few studies have addressed the association between drug overdose and HIV infection among IDUs. One study reported that IDUs with a history of drug overdose were more likely to have risky sexual behaviors [12], which increase the risk of HIV infection. Evidence on transmission risks can help guide interventions for HIV prevention. Therefore, we estimated HIV prevalence and attempted to identify factors associated with HIV infection among IDUs recruited at community-based methadone clinics in Taipei, Taiwan.

## Methods

### Study population and eligibility

This cross-sectional study consecutively recruited IDUs from Taipei City Hospital (TCH) methadone clinics from March 2012 to April 2013. The clinics were established in 2007 and now serve approximately 90.6% of methadone clients in Taipei [3]. All the clients are IDUs with a history of heroin addiction and are voluntarily enrolled in the program [3]. Each day they are required to report to methadone stands to receive free methadone therapy under direct supervision; those who fail to do so for 2 weeks or longer are removed from the therapy list. Our study included methadone clients who were 18 years of age or older, had a history of injecting illicit drugs, and had given written informed consent. Clients with severe cognitive deficits were excluded from this study. Study participants who completed the survey were compensated with 300 New Taiwan Dollars (equivalent of US$10) for their time. This study was approved by the Institutional Review Board of TCH (Approval number: TCHIRB-1000903).

### Data collection

At the time of study enrollment, consenting participants completed a face-to-face interview administered by trained case managers using a standardized questionnaire. The average duration of the interview was about 50 minutes. The case managers maintain a good rapport with participants, provide them with methadone therapy, monitor their treatment complications daily, and promote treatment adherence. The questionnaire was used to collect information on participant sociodemographics, substance use and injection practices, and sexual behaviors. Sociodemographic characteristics included age, gender, marital status, education completed, principal source of income during the previous 6 months, housing status during the previous 6 months, history of methadone treatment, and history and frequency of incarceration. Marital status was classified as unmarried, married, divorced, and widowed, and the categories for education level were elementary school or below and high school and above. Principal source of income during the previous 6 months was categorized as (1) regular work (defined as a full-time job; used as the reference category in statistical analysis), (2) temporary work or other noncriminal sources (ie, informal or temporary work, panhandling, social assistance, and financial support from families or friends), and (3) illegal sources (ie, theft, robbery, selling drugs, operating a drug injection site, and trading sex for money). Housing status was assessed by asking participants about the place in which they slept most often during the prior 6 months and was grouped into two broad categories: (1) stable housing (ie, living in their own house or apartment or that of their family, partner, or friend—the reference category) and (2) unstable housing (ie, living in workplaces, rented rooms, cars or other vehicles, abandoned buildings, shelters or welfare residences, jails, streets, or medical care organizations. The present participants included IDUs who were receiving methadone treatment and those who were seeking methadone therapy for the first time. History of methadone treatment was thus categorized as never, once, and twice or more.

Substance use and injection practices included age at first use of drugs, number of years of injecting, syringe and diluted water sharing during the previous 3 months, history of drug overdose (worded as "By drug overdose we mean taking enough drugs to stop your breathing or heartbeat"), history of cutaneous abscesses, and any alcohol use during the previous 3 months. The effect of police interference in drug use was investigated by asking participants, "How fearful are you that the police are going to arrest you or interfere with your drug use?". The possible responses to this question were "not at all", "somewhat", and "very". Sexual behaviors included age at sexual debut, ever giving someone money or drugs in exchange for sex, and history of sexually transmitted infection (STI).

### HIV testing

After the above interview, all participants had their blood drawn for HIV testing. HIV antibody testing was performed at the Kun-Ming Laboratory (Taipei, Taiwan) and consisted of an enzyme-linked immunosorbent assay (Genscreen HIV1/2 version 2) followed by Western blot confirmation of reactive samples, according to standard protocols [13].

### Statistical analysis

In bivariate analysis, variables were analyzed using means or medians for continuous measures and frequencies and percentages for categorical variables. The chi-square test was used to assess bivariate associations of selected factors with HIV infection. Multiple logistic regression analysis was used to estimate adjusted odds ratios (AORs) and their 95% confidence intervals (CIs) after controlling for other covariates. Analyses were conducted using the SPSS version 21.0 statistical software package (SPSS, Chicago IL, USA).

## Results

Of the 831 IDUs enrolled at the methadone program during the study period, 99.5% (827) completed both the interview and blood testing and were thus included in the subsequent analysis. Overall, the prevalence of HIV was 17.7% (95% CI 15.1–20.3%). The median age of the study subjects was 45 years (interquartile range 38–52 years); 85.9% were male; and the mean duration of injection drug use was 18.0 years (range 1–56 years).

In bivariate analysis, HIV-positive IDUs were more likely than HIV-negative IDUs to be aged ≤45 years, to be divorced, to have received most of their income from temporary jobs or other noncriminal sources during the previous 6 months (reference: regular jobs), and to have had more incarcerations (mean 3.0 vs. 2.6; p = 0.043) (Table 1). In terms of substance use and sexual behaviors, HIV-positive IDUs were more likely to have histories of overdose and STIs.

**Table 1:** Bivariate analysis of factors associated with HIV seropositivity among injection drug users at the methadone clinics in Taipei, Taiwan

**Factors**	**HIV (-) (n = 681)**	**HIV (+) (n = 146)**	**p value**	**OR (95% CI)**
**Sociodemographics**				
Age (years)				
18-45	349 (79.9)	88 (20.1)	0.047	1
>45	332 (85.1)	58 (14.9)		0.69 (0.48-0.99)
Gender				
Female	94 (80.3)	23 (19.7)	0.54	1
Male	587 (82.7)	123 (17.3)		0.86 (0.52-1.41)
Marital status				
Unmarried	333 (83.5)	66 (16.5)	0.024	1
Married	180 (87.0)	27 (13.0)		0.76 (0.47-1.23)
Divorce	159 (76.1)	50 (23.9)		1.59 (1.05-2.40)
Widowed	9 (75.0)	3 (25.0)		1.69 (0.44-6.38)
Education level completed				
≤Elementary school	110 (85.9)	18 (14.1)	0.246	1
High school and above	571 (81.7)	128 (18.3)		1.37 (0.80-2.34)
Source of most income during last 6 months				
Regular job	384 (85.3)	66 (14.7)	0.108	1
Temporal jobs or other legal source	270 (79.2)	71 (20.8)		1.53 (1.06-2.21)
Illegal source	27 (75.0)	9 (25.0)		1.94 (0.87-4.31)
Housing status during last 6 months				
Stable housing	434 (84.1)	82 (15.9)	0.087	1
Unstable housing	247 (79.4)	64 (20.6)		1.37 (0.96-1.97)
History of methadone treatment				
Never	49 (87.5)	7 (12.5)	0.143	1
Once	219 (85.2)	38 (14.8)		1.22 (0.51-2.88)
Twice or more	413 (80.4)	101 (19.6)		1.71 (0.75-3.89)
Number of incarceration				
Mean (range)	2.6 (0,10)	3.0 (0, 12)	0.043	1.09 (1.003-1.19)
**Substance use and injection practices**				
Age at first injection drug use (years)				
<20	93 (83.0)	19 (17.0)	0.837	1
≥20	588 (82.2)	127 (17.8)		1.06 (0.62-1.80)
Duration of drug use (years), mean (sd)	18.2 (10.4)	16.9 (8.0)	0.148	0.99 (0.97-1.01)
Shared syringes during last 3 months				
No	649 (82.2)	141 (17.8)	0.499	1
Yes	32 (86.5)	5 (13.5)		0.72 (0.28-1.88)
Shared diluted water during last 3 months				
No	633 (82.4)	135 (17.6)	0.836	1
Yes	48 (81.4)	11 (18.6)		1.08 (0.54-2.12)
History of drug overdose				
No	498 (84.7)	90 (15.3)	0.005	1
Yes	183 (76.6)	56 (23.4)		1.69 (1.17-2.46)
History of cutaneous abscess				
No	591 (83.0)	121 (17.0)	0.216	1
Yes	90 (78.3)	25 (21.7)		1.36 (0.84-2.20)
Any alcohol during last 3 months				
No	532 (83.0)	109 (17.0)	0.363	1
Yes	149 (80.1)	37 (19.9)		1.21 (0.80-1.84)
Very fearful of police interference during drug use				
No	288 (85.0)	51 (15.0)	0.101	1
Yes	393 (80.5)	95 (19.5)		1.37 (0.94-1.98)
**Sexual behaviors**				
Age at sexual debut (years)				
<16	122 (83.0)	25 (17.0)	0.820	1
≥16	559 (82.2)	121 (17.8)		1.06 (0.66-1.70)
Ever giving someone money in exchange for sex				
No	575 (81.8)	128 (18.2)	0.567	1
Yes	105 (85.4)	18 (14.6)		0.77 (0.45-1.32)
Unknown	1 (100.0)	0		(-)
History of sexually-transmitted infections				
No	574 (83.6)	113 (16.4)	0.044	1
Yes	107 (76.4)	33 (23.6)		1.57 (1.01-2.43)

Key: HIV = human immunodeficiency virus, OR = odds ratio, CI = confidence interval, STI = sexually transmitted infection.

Multiple logistic regression analysis showed that, after controlling for covariates, HIV infection was significantly associated with age ≤45 years (AOR = 1.62, 95% CI 1.01–2.62), being divorced (AOR = 1.67, 95% CI 1.06–2.62), deriving the majority of income from temporary jobs or other noncriminal sources during the previous 6 months (AOR = 1.53, 95% CI 1.02–2.30), unstable housing during the previous 6 months (AOR = 1.47, 95% CI 1.003–2.15), a higher number of incarcerations (AOR per incarceration =1.14, 95% CI 1.03–1.26), and a history of overdose (AOR = 1.51, 95% CI 1.01–2.28) (Table 2).

**Table 2:** **Multivariate logistic regression analysis of factors associated with HIV seropositivity among injection drug users at methadone clinics in Taipei, Taiwan**^**1**^

**Factors**	**AOR**	**95% CI**
Aged 18-45 years	1.65	1.02-2.67
Marital status		
Unmarried	1	
Married	0.92	0.55-1.55
Divorced	1.68	1.07-2.63
Widowed	2.10	0.53-8.37
Source of most income in last 6 months		
Regular job	1	
Temporal jobs or other legal source	1.59	1.05-2.39
Illegal source	1.65	0.70-3.87
Unstable housing in last 6 months	1.47	1.003-2.15
Number of incarceration (per time increase)	1.14	1.03-1.27
History of drug overdose	1.54	1.02-2.32

^1^after controlling for subject sociodemographics, substance use, and sexual behaviors.

Key: HIV = human immunodeficiency virus, AOR = adjusted odds ratio, CI = confidence interval.

## Discussion

Our analysis showed that the prevalence of HIV infection was 17.7% among IDUs at methadone clinics in Taipei during 2012–2013. After controlling for other characteristics, factors significantly associated with HIV infection were aged ≤45 years, being divorced, deriving the majority of income during the previous 6 months from temporary jobs or other noncriminal sources, unstable housing during the previous 6 months, a higher number of incarcerations, and a history of overdose.

As compared with IDUs at methadone programs in other Asian countries, HIV prevalence among IDUs was higher in Taipei than the 6% reported in China [14] but lower than the 35.9% reported in Thailand [11]. The high prevalence of HIV infection among IDUs in these countries is a serious public health problem that deserves increased attention. In 2006 the Taiwan CDC began a harm reduction program in response to a then rapidly growing HIV epidemic among IDUs. Currently there are 94 methadone clinics nationwide offering substitution treatment and clean syringes to IDUs (4). Consequently, estimated HIV incidence among IDUs in Taiwan decreased from 3.03/100 person-years at risk (PYAR) in 2005 to 0.93/100 PYAR in 2007 to 0.10/100 PYAR in 2012 (4).

In a comparison with previous findings on IDUs in Taiwan, HIV prevalence was higher in this study than the 12% noted among methadone clients in northern Taiwan in 2008 [9]. Also, 17.3% of male IDUs were HIV-positive in this study, which was lower than the rate of 25.5% among incarcerated male IDUs in 2005 [8]. The high prevalence of HIV among Taiwanese IDUs might be due to a change from smoked to injected heroin, which occurred during the most recent decade, [15] and/or to an increase in the frequency of HIV-associated risk behaviors such as needle sharing [10,16,17].

This study found that IDUs with a history of STIs had a higher prevalence of HIV infection than those without (23.6% vs. 16.4%). Furthermore, a history of overdose was associated with HIV infection in this study, after controlling for potential confounders. Previous study showed that IDUs with a history of overdose were more often involved in high-risk situations such as sex work [12], which might increase the risky behavior of unprotected sex. Since there is neither a cure nor a vaccine against HIV/AIDS, educating IDUs’ about HIV transmission and reducing their high-risk behaviors provide an important protection against the infection concerned [18,19].

This study also found that an age of ≤45 years was associated with HIV infection, which suggests that younger IDUs are involved in risky social networks [20] and thus more HIV-associated risk behaviors [21,22]. They also tend to lack knowledge of HIV infection [23], which could increase their risk of infection. Because the interventions that target the IDUs at highest risk are the most cost-effective measures [24], younger IDUs should be the focus of HIV prevention and education.

HIV prevalence was higher among IDUs with a greater number of incarcerations. Prior studies found that this IDU subgroup more often participated in injecting behaviors associated with HIV infection [25,26]. In addition, a cohort study in Taiwan showed that more than 40% of IDUs began using drugs again within 1.5 years after release from a correctional setting [27]. To better control the HIV epidemic among IDUs, incarcerated IDUs should receive education about HIV infection and, once released, should be strongly encouraged to refrain from drug use (eg, through harm reduction programs) [28].

Although not significant in the multivariable model, 58.5% of participants reported that they were "very" fearful of police interference in their drug use. Two qualitative studies showed that IDUs who perceive a threat of police interference during drug use will forgo safe injection practices to avoid unwanted interactions with police [29,30]. Further studies should assess the impact of policing on IDU injection practices.

As in prior studies, being divorced [31] and unstable housing [32] were associated with HIV infection.

The results of this study should be interpreted in the light of certain limitations. First, it is difficult to establish temporal sequences between factors investigated in cross-sectional research. Indeed, it is more difficult to do so in this study because HIV-positive patients often do not know exactly when they were infected and because we have no information on the HIV status of study subjects before their participation in this study. However, the HIV status of the participants was evaluated by laboratory testing rather than by self-report. Those found to be HIV-positive were referred to HIV specialists for further evaluation and treatment. Second, information bias regarding risk behaviors cannot be avoided by using a self-reported questionnaire. We reduced this bias by having the interviews conducted by the case managers—that is to say, the same people who provided the study subjects with methadone treatment and psychological support. Third, it is difficult to obtain a truly representative sample of a population of community-dwelling IDUs. It was estimated that methadone clients accounted for 10.8% of reported heroin-addicted drug users in Taipei in 2012 [33]. Thus, the generalizability of the present findings might be limited to IDUs at Taipei methadone clinics.

## Conclusions

In conclusion, Taiwanese IDUs at methadone clinics had a relatively high HIV prevalence, which was associated with younger age and history of overdose. It is imperative to educate IDUs’ about HIV transmission, particularly for the younger and overdosed IDUs.

## Competing interests

The authors declare that they have no competing interests.

## Author contributions

YFY, MYY, TL, PC, and CYD substantially contributed to the conception and design of the study, data analysis, data interpretation, and the drafting of the manuscript. LHL and XRJ substantially contributed to data acquisition and interpretation of the results. YFY, MYY, TL, LHL, XRJ, PC, and CYD all approved the final version of the manuscript.

## Pre-publication history

The pre-publication history for this paper can be accessed here:

http://www.biomedcentral.com/1471-2458/14/682/prepub
